# Supplementary material for: Mouse brain transcriptome responses to inhaled nanoparticulate matter differed by sex and APOE in Nrf2-Nfkb interactions
Source: eLife. 2020 Jun 24;9:e54822. doi: 10.7554/eLife.54822 (PMC7314548; doi:10.7554/eLife.54822)
Supplement: Supplementary file 1. [file elife-54822-supp1.docx]

Table S1. qPCR Primers used in this study.

| Gene | Forward | Reverse |
| --- | --- | --- |
| Nfe2l1 | TCTGGCAGTATCTGGAACTT | GGGGTTTATAGGCTTTTGTT |
| mTor | ACCTGGATGACTATCTGCAC | AACTATTGGGTGAATGATGC |
| B2m | TATGCCAAACCCTCTGTACT | AAAAGCAGAAGTAGCCACAG |
| Foxo3 | GGAACTTCACTGGTGCTAAG | CTCTGTAGGTCTTCCGTCAG |
| Apoc1 | GCAAAGTGAAGGAGAAGTTG | ATTGGTCTGTGATGAAGAGG |
| Sirt1 | GTTTGTACCACCAAATCGTT | ATGTGCCACTGTCACTGTTA |
| Nqo2 | GTGAAACACAGGGATTAGGA | GGGAAGGTCTCATGTAACAA |
| Tap1 | CTATCAGTTATGTGGCAGCA | CAAGGCAAGAGAGAATCAAG |
| Eomes | GCTTCAACATAAACGGACTC | GCCAGTGTTAGGAGATTCTG |
| Ddx3y | GACATGATGGAAAGAGGAAA | GAGCAAGCATCTGTATCTCC |
| Xist | CAAGTGTGAAAGTGTTGGTG | TCCTTATGGGACAGTGACTC |
| Erdr1 | TGCCCTAATTATTCTTGTCC | GGTTAGACTTTCCATTCACG |
| Gapdh | CCAATGTGTCCGTCGTGGATCT | GTTGAAGTCGCAGGAGACAACC |
| NF-κB1 | CCAGAAGAGGGTGTCAGAGC | ACATTTGCCCAGTTCCGTAG |
| Nfe2l2 | CATAGAGCAGGACATGGAGCAA | TCCATTTCTGTCAGTGTGGCTT |
| IL1β | CTAAAGTATGGGCTGGACTG | GGCTCTCTTTGAACAGAATG |
| IL6 | TGCCTTCTTGGGACTGATGCT | GCATCCATCATTTCTTTGTAT |
| NOS2 | GTCTTGGTGAAAGTGGTGTT | GTGCTTGCCTTATACTGGTC |
| RelA | GCGTACACATTCTGGGGAGT | ACCGAAGCAGGAGCTATCAA |
